# The differing relationships between academic discipline, sleep hygiene, and dysfunctional sleep attitudes on sleep quality and duration in Canadian university students

**DOI:** 10.3389/fpsyg.2024.1396579

**Published:** 2024-08-02

**Authors:** Tara Kuhn, Sameena Karsan, Jennifer J. Heisz, Laura E. Middleton

**Affiliations:** ^1^Brain and Body Lab, Department of Kinesiology and Health Sciences, University of Waterloo, Waterloo, ON, Canada; ^2^Neurofit Lab, Department of Kinesiology, McMaster University, Hamilton, ON, Canada

**Keywords:** sleep, insomnia, sleep hygiene, health knowledge, attitudes, practice, academic discipline, mental health

## Abstract

**Objectives:**

This study sought to understand how university student’s academic discipline relates to sleep factors including attitudes, hygiene, quality, and duration.

**Methods:**

Using a cross-sectional approach, a 30-min survey was advertised to students at two Canadian universities in March of 2022. Sleep measures included the Dysfunctional Beliefs and Attitudes Scale, the Sleep Hygiene Index, the Pittsburgh Sleep Quality Index, and the Insomnia Severity Index. Academic discipline was categorized into four groups: Health, Arts, Sciences, and Engineering. Multiple linear regressions were used to investigate (1) the effect of academic discipline on sleep measures, and (2) the effect of academic discipline, dysfunctional sleep attitudes, and sleep hygiene practices on insomnia, sleep quality and duration.

**Results:**

1,566 students completed the survey (69.4% women; 80.3% undergraduates). Compared to Health students, Art students had worse dysfunctional sleep attitudes, hygiene, quality, and insomnia severity (*p* < 0.001). This relationship disappeared after controlling for differences in mental health (*p ≥* 0.05). Art students had longer sleep durations (*p* < 0.01), whereas Engineering students had shorter sleep durations (*p* < 0.05). When dysfunctional sleep attitudes and hygiene were included in the model, both academic discipline (*p* < 0.05) and sleep hygiene (*p* < 0.001) were associated with sleep duration. Sleep hygiene and attitudes were also associated with sleep quality and insomnia severity (*p* < 0.001), while academic discipline was not (*p ≥* 0.05).

**Discussion:**

These results suggest differences in sleep quality across academic discipline are impacted by dysfunctional sleep attitudes, sleep hygiene, and mental health, whereas differences in sleep duration appear to be independent of these factors. Overall, students in different academic disciplines have unique relationships with sleep and thus may require targeted approaches to improve their sleep. Future interventions should focus on supporting sleep hygiene and attitudes to improve students’ sleep and well-being.

## 1 Introduction

Poor sleep can have negative consequences for university students. Nearly one-third of university students do not get the recommended 8–10 h of sleep (Hirshkowitz et al., 2015; Wang et al., 2016; Norbury and Evans, 2018; Humphries et al., 2021; Ali et al., 2023), and approximately half report having poor sleep quality, though estimates vary considerably across studies (Suen et al., 2008; Lund et al., 2010; Lopes et al., 2013; Rezaei et al., 2018; Rao et al., 2020; Humphries et al., 2021; Alamir et al., 2022). Further, a meta-analysis estimated that 18.5% of university students report experiencing insomnia (Jiang et al., 2015). Poor sleep is associated with worse mental health, including higher levels of stress and increased odds of having depression or anxiety (Lund et al., 2010; Haregu et al., 2015; Norbury and Evans, 2018; Gardani et al., 2022). Furthermore, students who sleep less than the recommended hours per night tend to have worse academic performance (Curcio et al., 2006; Gaultney, 2010; Gomes et al., 2011). Similarly, students who report poor sleep quality have poorer academic performance, on average (Gaultney, 2010; Gilbert and Weaver, 2010; Gomes et al., 2011; Lopes et al., 2013; Cates et al., 2015; Al-Kandari et al., 2017; Seoane et al., 2020; Suardiaz-Muro et al., 2020; Turner et al., 2021). Difficulties falling asleep are a prominent complaint among this population (Jensen, 2003; Lund et al., 2010; Wang et al., 2016; Humphries et al., 2021).

Various factors contribute to university students’ poor sleep. Firstly, university students often have many competing demands. Students may sacrifice sleep to meet these competing demands, which can hinder their physical, emotional, and cognitive health (Curcio et al., 2006; Vail-Smith et al., 2009; Lund et al., 2010). In addition, university life can be stressful for young adults. Many students report sleep difficulties as a result of academic stress or worrying (e.g., about their future; Lund et al., 2010; Humphries et al., 2021). Furthermore, students report worrying or feeling helpless about their ability to get enough sleep, which can further worsen sleep quality and insomnia severity, including delaying sleep onset (Brown et al., 2002; Harvey, 2002; Suen et al., 2008; Jin et al., 2018). This highlights the potential bi-directional relationship between sleep and mental health, where poor mental health can contribute to poor sleep and vice versa, poor sleep can negatively impact mental health (Alvaro et al., 2013; Fang et al., 2019).

Students’ sleep hygiene practices and attitudes toward sleep may also contribute to their poor sleep. Sleep hygiene refers to the various behavioral and environmental practices used to improve sleep – for example, avoiding the use of electronic devices before bed and ensuring the bedroom is cool, dark, and quiet. Better sleep hygiene is associated with better sleep quality and longer sleep durations (Brown et al., 2002; Revathi et al., 2016; Ruggiero et al., 2019; Ali et al., 2023).

Dysfunctional sleep attitudes relate to one’s unrealistic expectations, worries, or fears about their sleep requirements and the potential consequences of failing to meet them (Morin et al., 2007). These dysfunctional attitudes and beliefs can lead to worry and stress, resulting in hyperarousal and perpetuating insomnia (Harvey, 2002). Further, dysfunctional attitudes about sleep are associated with poorer sleep or greater insomnia severity in university students (Yang and Chou, 2011; Calkins et al., 2012; Doos Ali Vand et al., 2014; Jin et al., 2018); though not all studies show agreement (Joshi et al., 2015; Humphries et al., 2021). The discrepancy in findings may be due to differences in study samples. Most sleep studies examined relatively homogenous student groups – for example, first-year psychology students (Calkins et al., 2012; Humphries et al., 2021), pharmacy students (Cates et al., 2015), or medical students (Joshi et al., 2015; Wang et al., 2016; Rezaei et al., 2018). For example, one study failed to find a relationship between dysfunctional sleep attitudes and sleep quality or duration, but they only tested first-year psychology students (Humphries et al., 2021). Another study compared medical students with non-medical students and found that dysfunctional sleep attitudes, but not sleep quality, were significantly better in the medical students (Jin et al., 2018). Jin et al. (2018) speculated medical education may correct erroneous attitudes about sleep, suggesting that the influence of dysfunctional sleep attitudes may vary depending on academic discipline.

No research has directly examined how academic discipline impacts sleep, dysfunctional sleep attitudes, and sleep hygiene practices across a broad range of disciplines. Further, given the bi-directional relationship between sleep and mental health, it is important to also consider mental health when examining these associations. Therefore, the objective of this study was to examine how academic discipline is associated with sleep, dysfunctional sleep attitudes, and sleep hygiene among university students while controlling for mental health. A secondary objective was to examine the combined effect of academic discipline, dysfunctional sleep attitudes, and sleep hygiene on sleep quality and duration. It was hypothesized that students in health disciplines would have a better relationship with sleep, as health-related studies may have similar positive sleep attitudes and consequently sleep benefits as previously seen with medical education (Jin et al., 2018).

## 2 Methods

### 2.1 Study design and setting

This cross-sectional survey was conducted among students at the University of Waterloo and McMaster University in Ontario, Canada. The study was approved by institutional research ethics committees (University of Waterloo ORE #43903, McMaster University MREB #5834). All participants provided electronic informed consent. Data collection occurred between March 7 to 25th 2022.

### 2.2 Participants

Participants only needed to report current enrollment as a student at either institution to take part in the study; there were no other inclusion or exclusion criteria. Only completed surveys were included in this analysis. Students were recruited through a variety of strategies, including posters, social media posts, McMaster’s Sona system, and advertisements in email bulletins.

### 2.3 Measures

#### 2.3.1 Demographics and covariates

Demographic information included age, gender, sex, household income, ethnicity, and diagnosis of sleep disorders or psychiatric conditions. Participants reported their student status, including their year, study level (undergraduate or graduate), department, and faculty. Questions also probed further details about their sleep practices, including “*Do you perceive your academic studies to be health-orientated or health-focused?”* and “*Have you ever pulled an “all-nighter” (voluntarily skipping sleep and staying up for nearly 24 h or greater) during your academic career?.”* There were also several questionnaires used to characterize mental health, including stress (perceived stress scale, PSS; Cohen et al., 1983), depression (Patient Health Questionnaire, PHQ-9; Kroenke et al., 2001), and anxiety (General Anxiety Disorder scale, GAD-7; Spitzer et al., 2006). Across all mental health measures, a higher score indicated more severe mental distress.

#### 2.3.2 Academic discipline

Students reported their faculty using a drop-down menu and recorded which department they were in using an open textbox. Based on the participant’s faculty and department, students were categorized into four academic disciplines: Arts (including Fine Arts, Social Sciences, and Humanities), Sciences (including Math), Health, and Engineering. There was good agreement between universities for which departments were in each faculty, except for kinesiology and computer science. For this study, we categorized kinesiology as “Health” and computer science as “Engineering.”

#### 2.3.3 Dysfunctional sleep attitudes

To assess dysfunctional sleep attitudes, the *Dysfunctional Beliefs and Attitudes about Sleep Scale* (DBAS-16; Morin et al., 2007) was used. Participants recorded their agreement with 16 statements on a Likert scale, with 0 indicating “strongly disagree” and 10 indicating “strongly agree”; a higher score indicated more dysfunctional attitudes and beliefs. A global score was calculated by taking the mean of the summed items. The DBAS has adequate internal consistency in a sample of patients in a sleep clinic (Cronbach alpha =0.77; Morin et al., 2007). A DBAS score greater than 3.5 indicates clinically significant dysfunctional sleep attitudes (Smith and Trinder, 2001; Carney et al., 2010).

#### 2.3.4 Sleep quality

The *Pittsburgh Sleep Quality Index* (PSQI) was used to measure subjective sleep quality and sleep duration (Buysse et al., 1989). The PSQI asks participants about their sleep habits during the last month within seven components: subjective sleep quality, sleep latency, sleep duration, sleep efficiency, difficulties sleeping, use of sleep medications, and sleepiness. Participants scores are categorized into the frequency of sleep disturbances (0 = least frequent, 3 = most frequent), for a maximum score of 21. A higher score is indicative of greater sleep disturbance, and a score above five indicates a “poor sleeper” (Buysse et al., 1989). The PSQI has good internal consistency (Cronbach alpha = 0.83), with good sensitivity (89.6%) and specificity (86.5%) in discriminating between “good” and “poor” sleepers (Buysse et al., 1989).

#### 2.3.5 Sleep hygiene

Sleep hygiene was assessed using the *Sleep Hygiene Index* (SHI; Mastin et al., 2006). This 13-item scale asks participants to evaluate the frequency of specific sleep hygiene behaviors, with 0 indicating “never” and 4 indicating “always.” A global score is calculated by taking the sum, with a minimum score of 0 and a maximum of 52, and a higher score indicating poorer sleep hygiene. This scale has reliable internal consistency (Cronbach alpha = 0.66) and good test–retest reliability [*r*(139) = 0.71, *p* < 0.01] in university students (Mastin et al., 2006).

#### 2.3.6 Insomnia severity

Insomnia was assessed using the *Insomnia Severity Index* (ISI; Bastien et al., 2001). This is a seven-item questionnaire which asks participants about the nature, severity, and the impact of insomnia. Participants’ scores indicate the severity of each insomnia symptom (0 = no problem; 4 = severe problem), and a higher score indicates greater insomnia severity. In Canadian adults, the ISI has very good internal consistency in community samples (Cronbach alpha = 0.90; Morin et al., 2011).

### 2.4 Statistical analyses

Statistical analyses were completed using *R* computing software (R Core Team, 2023). Data were screened for missing and extreme values. Missing data was addressed by mean imputation or single-case removal. For mental health measures, insomnia, and sleep hygiene, having two or fewer missing values was resolved using mean imputation (*n* = 14; Kroenke et al., 2010), whereas total scores were removed when there were more than two missing values (*n* = 6). PSQI global scores were removed if any data was missing, as certain component scores cannot be estimated or imputed (e.g., sleep duration, sleep latency; *n* = 21). Data were screened for extreme values, and participants were removed if they were suspected of inserting random responses (e.g., a value outside of the accepted range for multiple measures; *n =* 11).

For all analyses, the reference category for academic discipline was Health. Health was selected as the reference group, as it is the discipline most similar to medical studies, which has been suggested to be protective against incorrect perceptions of sleep (Jin et al., 2018). Demographic characteristics as well as sleep and mental health indicators were compared across disciplines using chi-squared tests for categorical variables and one-way ANOVAs with post-hoc, pairwise t-tests for continuous variables. For all analyses, the alpha level was 0.05.

#### 2.4.1 Primary objective

To investigate the independent effect of academic discipline on sleep (PSQI global score, sleep duration, insomnia severity), dysfunctional sleep attitudes (DBAS score) or sleep hygiene (SHI score), hierarchical multiple linear regressions were conducted. The unadjusted model contained academic discipline with no other covariates. The base model contained academic discipline and key demographic covariates (age, sex, income, university). To account for the potential confounding effect of mental health, the fully adjusted model included academic discipline, demographic covariates, and assessments of mental health (stress, anxiety, and depression).

#### 2.4.2 Secondary objective

Hierarchical linear regression models were used to investigate the relationships between academic discipline, dysfunctional sleep attitudes, and sleep hygiene practices in relation to sleep indicators. The unadjusted model included academic discipline, dysfunctional sleep attitudes, and sleep hygiene. Then, demographic covariates were included in the base model, and mental health measures were added to the fully adjusted model.

## 3 Results

1,597 students completed the survey. After screening for completeness, 18 duplicate responses were removed (indicated by Sona identification number), two were removed because they did not meet eligibility criteria (one had graduated; one was staff), and 11 were removed for extreme values across multiple sleep and psychological measures, for a final sample of 1,566.

### 3.1 Participant characteristics

See Table 1 for demographic characteristics. There was roughly equal representation from both institutions across all disciplines. There were more women and undergraduate students, and less than half of the students identified as white or Caucasian. Additionally, nearly two-thirds of the sample were considered poor sleepers, over one-third were not getting 7 h of sleep, and 3% reported being diagnosed with a sleep disorder. When looking at academic disciplines, the unadjusted one-way ANOVAs revealed that Art students had more severe dysfunctional attitudes (*p* < 0.001), and worse sleep quality, sleep hygiene practices, and insomnia severity (*p* < 0.01). Arts students also reported having higher levels of depression (*p* < 0.001), stress and anxiety (*p* < 0.01). Further, Science students had more dysfunctional attitudes and higher levels of depression and stress compared to Health students (*p* < 0.01). Engineering students were not significantly different to Health students in most mental health and sleep measures, except for having shorter sleep durations and lower anxiety compared to Health students (*p <* 0.05).

**Table 1:** Participant demographics (mean [standard deviation] or n [%]).

	All disciplines	Health	Arts	Engineering	Sciences	*p* ^†^
*n*	1,566	310 (20%)	384 (24.5%)	484 (31%)	388 (24.8%)	
Age	21.77 (4.82)	22.36 (6.02)	22.31 (5.59)	21.60 (4.18)*	20.99 (3.39)***	<0.001
Institution, Waterloo	732 (46.7%)	151 (48.7%)	192 (50%)	226 (46.7%)	163 (42.0%)	0.14
Institution, McMaster	834 (53.3%)	159 (51.3%)	192 (50%)	258 (53.3%)	225 (58.0%)	0.14
Sex, female	1,123 (71.7%)	271 (87.4%)	318 (82.3%)	245 (50.6%)	291 (75.0%)	<0.001
Gender, women	1,087 (69.4%)	268 (86.5%)	303 (78.9%)	237 (49.0%)	279 (71.9%)	<0.001
Race/Ethnicity, white/Caucasian	655 (41.8%)	143 (46.1%)	193 (50.2%)	173 (35.7%)	146 (36.6%)	<0.001
Health related studies	756 (48.3%)	300 (96.8%)	119 (31%)	127 (26.2%)	210 (54.1%)	<0.001
Level of studies, undergraduate	1,258 (80.3%)	229 (73.9%)	310 (80.7%)	409 (84.5%)	310 (79.9%)	0.01
Diagnosed with a mental health disorder	46 (2.9%)	9 (2.9%)	15 (3.9%)	10 (2.1%)	12 (31%)	<0.001
Diagnosed with a sleep disorder, yes	278 (17.8%)	50 (16.1%)	104 (27.1%)	62 (12.8%)	62 (16.0%)	<0.001
Completed an all-nighter	902 (57.6%)	161 (51.9%)	238 (62.0%)	278 (57.4%)	255 (58.0%)	0.14
“Poor” Sleepers (PSQI >5)	1,046 (67.7%)	191 (62.6%)	284 (74.9%)	314 (65.1%)	257 (67.8%)	0.01
≥ 7 h of sleep	969 (62.1%)	192 (61.9%)	261 (68.1%)	290 (59.9%)	226 (59.0%)	0.04
Depression (PHQ)	9.49 (6.33)	8.64 (6.17)	10.70 (6.31)***	8.61 (6.03)	10.07 (6.61)**	<0.001
Stress (PSS)	20.85 (6.71)	20.19 (6.43)	21.75 (6.44)**	19.78 (6.89)	21.83 (6.70)**	<0.001
Anxiety (GAD-7)	9.12 (5.68)	8.99 (5.57)	10.19 (5.60)**	8.10 (5.55)*	9.44 (5.79)	<0.001
Dysfunctional sleep attitudes (DBAS)	4.70 (1.52)	4.47 (1.47)	4.87 (1.58)***	4.65 (1.49)	4.78 (1.51)**	0.003
Sleep quality (PSQI global score)	7.31 (3.23)	6.97 (3.20)	7.77 (3.19)**	7.08 (3.21)	7.42 (3.28)	0.003
Sleep duration (in hours)	6.95 (1.18)	6.99 (1.22)	7.17 (1.22)*	6.81 (1.13)*	6.86 (1.13)	<0.001
Sleep hygiene (SHI)	23.65 (6.62)	23.24 (6.55)	24.93 (6.63)**	22.97 (6.34)	23.54 (6.83)	<0.001
Insomnia severity (ISI)	9.67 (7.72)	9.14 (5.83)	10.41 (5.78)**	9.25 (5.45)	9.89 (5.84)	0.007

^†^*p*-values are based on 1-way ANOVA or chi-squared test; Differences in age, depression, stress, and anxiety were determined using a t-test using Health as the reference;*p < 0.05, **p < 0.01, ***p < 0.001; DBAS, Dysfunctional Beliefs and Attitudes about Sleep Scale; PSQI, Pittsburgh Sleep Quality Index; SHI, Sleep Hygiene Index; ISI, Insomnia Severity Index; PHQ, Patient Health Questionnaire; PSS, Perceived Stress Scale; GAD-7, General Anxiety Disorder scale.

### 3.2 Associations between academic discipline and dysfunctional sleep attitudes, sleep hygiene, and other sleep measures

When looking at the relationship between academic discipline and dysfunctional sleep attitudes, sleep hygiene, or other measures of sleep (Table 2), all academic disciplines had more dysfunctional sleep attitudes compared to Health students, but this difference disappeared after accounting for mental health. Art students had worse sleep hygiene habits (*p* < 0.001), sleep quality (*p* < 0.01), and insomnia severity (*p* < 0.01), compared to Health students but this effect disappeared after controlling for mental health. Engineering students had the shortest sleep duration in both the base model (*p* < 0.05) and when accounting for mental health (*p* < 0.05), while Art students had significantly longer sleep durations only when accounting for mental health (*p* < 0.01).

**Table 2:** Regression coefficients for the independent association of academic discipline on sleep, dysfunctional sleep attitudes, and sleep hygiene.

	Unadjusted model*			Base Model**			Full model***		
	*b* (95% CI)	*R^2^*	*p*	*b* (95% CI)	*R^2^*	*p*	*b* (95% CI)	*R^2^*	*p*
Dysfunctional Sleep Attitudes (DBAS)									
		0.01	0.003		0.01	**<0.001**		0.18	**<0.001**
Health	Ref		–	Ref		–	Ref		–
Arts	0.4 (0.17, 0.63)		**<0.001**	0.42 (0.19, 0.65)		**<0.001**	0.21 (0, 0.42)		0.05
Engineering	0.18 (−0.04, 0.4)		0.1	0.24 (0.01, 0.47)		**0.04**	0.2 (−0.01, 0.41)		0.06
Sciences	0.31 (0.08, 0.53)		**< 0.01**	0.32 (0.09, 0.55)		**< 0.01**	0.18 (−0.03, 0.4)		0.09
Sleep Hygiene (SHI)									
		0.01	**<0.001**		0.06	**<0.001**		0.37	**<0.001**
Health	Ref		–	Ref		–	Ref		–
Arts	1.7 (0.71, 2.68)		**<0.001**	1.82 (0.84, 2.8)		**<0.001**	0.6 (−0.22, 1.41)		0.15
Engineering	−0.27 (−1.21, 0.67)		0.58	−0.11 (−1.08, 0.86)		0.82	−0.28 (−1.08, 0.53)		0.5
Sciences	0.3 (−0.68, 1.29)		0.54	0.01 (−0.97, 0.99)		0.98	−0.71 (−1.53, 0.1)		0.08
Sleep Quality (PSQI Global score)									
		0.01	0.003		0.02	**<0.001**		0.40	**<0.001**
Health	Ref		–	Ref		–	Ref		–
Arts	.79 (0.31, 1.28)		**0.001**	0.82 (0.33, 1.31)		**0.001**	0.16 (−0.23, 0.55)		0.42
Engineering	0.11 (−0.35, 0.57)		0.63	0.20 (−0.22, 0.76)		0.28	0.18 (−0.20, 0.57)		0.34
Sciences	0.45 (−0.03, 0.10)		0.24	0.44 (−0.05, 0.94)		0.08	0.07 (−0.32, 0.46)		0.72
Self-Reported Sleep Duration									
		0.01	**<0.001**		0.02	**<0.001**		0.10	**<0.001**
Health	Ref		–	Ref		–	Ref		–
Arts	0.18 (0, 0.35)		0.05	0.17 (0, 0.35)		0.06	0.27 (0.10, 0.45)		**< 0.01**
Engineering	−0.18 (−0.35, −0.01)		**0.04**	−0.21 (−0.38, −0.03)		**0.02**	−0.19 (−0.36, −0.02)		**0.03**
Sciences	−0.13 (−0.30, 0.5)		0.15	−0.11 (−0.29, 0.07)		0.23	−0.04 (−0.22, 0.13)		0.63
Insomnia Severity (ISI)									
		0.01	0.01		0.03	**<0.001**		0.49	**<0.001**
Health	Ref		–	Ref		–	Ref		–
Arts	1.27 (0.31, 2.23)		**< 0.01**	1.30 (0.44, 2.17)		**< 0.01**	0.03 (−0.61, 0.66)		0.94
Engineering	0.11 (−0.71, 0.92)		0.79	0.44 (−0.42, 1.30)		0.32	0.31 (−0.32, 0.93)		0.34
Sciences	0.75 (−0.11, 1.60)		0.08	0.70 (−0.17, 1.57)		0.11	−0.06 (−0.70, 0.58)		0.86

*Adjusted for academic discipline; **Adjusted for age, sex, income, and university; ***Adjusted for age, sex, income, university, depression, anxiety, and stress. DBAS, Dysfunctional Beliefs and Attitudes about Sleep Scale; PSQI, Pittsburgh Sleep Quality Index; SHI, Sleep Hygiene Index; ISI, Insomnia Severity Index.

### 3.3 Associations between academic discipline, dysfunctional sleep attitudes, sleep hygiene with sleep quality, duration, and insomnia severity

Academic discipline was only associated with sleep duration (Table 3), where Arts students had more sleep (*p* < 0.01) and Engineering students (*p* < 0.05) had less sleep than Health students when controlling for dysfunctional sleep attitudes and sleep hygiene behavior. In contrast, dysfunctional sleep attitudes were associated with worse sleep quality and insomnia severity (*p* < 0.001). Poor sleep hygiene practices were associated with worse sleep quality (*p* < 0.001), shorter sleep durations (*p* < 0.05), and greater insomnia severity (*p* < 0.001).

**Table 3:** Regression coefficients for association between sleep quality and duration by academic discipline, dysfunctional sleep attitudes, and sleep hygiene.

		Unadjusted model*			Base Model**			Full model***		
		*b* (95% CI)	*R^2^*	*p*	*b* (95% CI)	*R^2^*	*p*	*b* (95% CI)	*R^2^*	*p*
Sleep Quality (PSQI Global score)										
			0.33	**<0.001**		0.34	**<0.001**		0.46	**<0.001**
DBAS		0.72 (0.63, 0.81)		**<0.001**	0.7 (0.61, 0.8)		**<0.001**	0.46 (0.37, 0.55)		**<0.001**
SHI		0.18 (0.16, 0.2)		**<0.001**	0.19 (0.17, 0.21)		**<0.001**	0.08 (0.06, 0.11)		**<0.001**
Academic discipline										
	Health	Ref		–	Ref		–	Ref		–
	Arts	0.21 (−0.19, 0.61)		0.31	0.18 (−0.23, 0.59)		0.38	0.01 (−0.36, 0.39)		0.94
	Engineering	0.03 (−0.35, 0.41)		0.89	0.11 (−0.3, 0.51)		0.6	0.12 (−0.25, 0.48)		0.54
	Sciences	0.19 (−0.21, 0.59)		0.34	0.22 (−0.19, 0.63)		0.29	0.05 (−0.32, 0.42)		0.8
Self-Reported Sleep Duration										
			0.04	**<0.001**		0.07	**<0.001**		0.12	**<0.001**
DBAS		0 (−0.04, 0.04)		1	0 (−0.04, 0.04)		0.93	0.06 (0.02, 0.1)		**< 0.01**
SHI		−0.04 (−0.05, −0.03)		**<0.001**	−0.04 (−0.05, −0.03)		**<0.001**	**−**0.01 (−0.02, 0)		**0.03**
Academic discipline										
	Health	Ref		–	Ref	–	Ref	Ref	–	–
	Arts	0.24 (0.07, 0.41)		**0.01**	0.24 (0.07, 0.42)		**0.01**	0.28 (0.11, 0.45)		**< 0.01**
	Engineering	−0.19 (−0.21, −0.59)		**0.02**	−0.21 (−0.38, −0.04)		**0.02**	−0.21 (−0.38, −0.04)		**0.02**
	Sciences	−0.12 (−0.29, 0.06)		0.18	−0.11 (−0.29, 0.07)		0.22	−0.06 (−0.24, 0.11)		0.46
Insomnia Severity (ISI)										
			0.44	**<0.001**		0.46	**<0.001**		0.59	**<0.001**
DBAS		1.61 (1.47, 1.76)		**<0.001**	1.61 (1.46, 1.76)		**<0.001**	1.14 (1, 1.27)		**<0.001**
SHI		0.34 (0.31, 0.38)		**<0.001**	0.35 (0.31, 0.38)		**<0.001**	0.15 (0.11, 0.18)		**<0.001**
Academic discipline										
	Health	Ref	–	–	Ref	–	–	Ref		–
	Arts	0.05 (−0.6, 0.7)		0.88	0.01 (−0.65, 0.67)		0.98	−0.31 (−0.89, 0.26)		0.28
	Engineering	−0.08 (−0.7, 0.53)		0.8	0.11 (−0.54, 0.75)		0.75	0.11 (−0.46, 0.67)		0.71
	Sciences	0.15 (−0.5, 0.79)		0.66	0.18 (−0.47, 0.84)		0.59	−0.18 (−0.75, 0.39)		0.54

*Models contain DBAS, SHI, and academic discipline; **Adjusted for age, sex, income, and university; ***Adjusted for age, sex, income, university, depression, anxiety, and stress. DBAS, Dysfunctional Beliefs and Attitudes about Sleep Scale; PSQI, Pittsburgh Sleep Quality Index; SHI, Sleep Hygiene Index; ISI, Insomnia Severity Index.

## 4 Discussion

This study investigated the impact of academic discipline on the association between sleep, dysfunctional sleep attitudes, and sleep hygiene. It also examined how dysfunctional sleep attitudes and hygiene relate to sleep quality and duration. Art students had the poorest sleep of all academic disciplines, scoring worse in sleep quality, sleep hygiene, and dysfunctional sleep attitudes, but not when controlling for mental health. Although academic discipline significantly predicted sleep quality and insomnia severity in the baseline models, these effects disappear when considering mental health, dysfunctional sleep attitudes, and sleep hygiene. This suggests that academic discipline may influence sleep quality and insomnia severity through these factors but does not have an independent effect. However, academic discipline was significantly associated with sleep duration across all models, suggesting an independent relationship.

Overall, students’ sleep attitudes and sleep quality were poor, as evident by clinically significant dysfunctional sleep attitudes (scores >3.5) and high PSQI global scores (scores >5). Similar to other studies, nearly two-thirds of our sample were considered “poor sleepers” as revealed by the PSQI global score (Suen et al., 2008; Lund et al., 2010; Lopes et al., 2013; Rezaei et al., 2018; Humphries et al., 2021) and one-third failed to meet the recommended 7 h of sleep (Hirshkowitz et al., 2015; Norbury and Evans, 2018; Humphries et al., 2021). These results are extremely concerning. Sleep is critical for students’ physical and mental well-being (Lund et al., 2010; Haregu et al., 2015; Norbury and Evans, 2018; Carpi et al., 2022) as well as their academic success (Seoane et al., 2020; Suardiaz-Muro et al., 2020; Turner et al., 2021). The observation that in general, students across four academic disciplines and two universities have extremely dysfunctional attitudes about sleep and are not sleeping well highlights a critical need for interventions supporting student’s sleep needs, which will be discussed further below.

Across the disciplines, Art students consistently scored poorly across sleep quality metrics (i.e., PSQI global score and insomnia severity), possibly driven by poor mental health, dysfunctional sleep attitudes, and sleep hygiene, all of which were also worst among Arts students. Indeed, other studies have also found higher rates of mental illness among Art students (Lipson et al., 2016; Allen et al., 2022), as they tend to suffer more from depression, anxiety, and bipolar disorder (McLafferty et al., 2022). Why do Art students have the poorest sleep and mental health of all students? The answer is likely multifaceted and requires further investigation.

Contrary to expectations, Engineering students had comparable mental health and sleep quality—albeit shorter sleep durations—to Health students. Engineering is reputable for its intensity: high course loads, high volume of assignments, and challenging concepts, all acting as potential stressors for students (Jensen et al., 2023). Although some studies find Engineering students have high mental distress (Leahy et al., 2010), others have found similar results to the present study (Lipson et al., 2016; Allen et al., 2022). We speculate on several reasons for this discrepancy in our study. First, Engineering students who are overwhelmed with their workload may not participate in a 20–30-min survey, and thus the sample may reflect students with the capacity to participate. Second, the questionnaires were self-reported, and their answers may have been skewed to reflect an engineering culture that values grit, rigor, and toughness (Rohde et al., 2020; Sochacka et al., 2021) and normalizes (and even dismisses) stress and mental illness (Beddoes and Danowitz, 2022; Jensen et al., 2023). This may also explain why Engineering students had similar sleep quality but reported the shortest sleep durations: functioning on little sleep may be acceptable in a culture of toughness and resiliency. Finally, the Engineering students in our study were predominately men, which may have impacted the results in several ways. One potential reason is that certain sleep characteristics are better in men (e.g., shorter sleep latencies; Mallampalli and Carter, 2014). Additionally, men tend to report lower mental distress, which historically has been attributed to resiliency, but may reflect biases in diagnostic criteria producing false negatives or a social desirability bias whereby men may feel they need to be strong and stoic (Whitley, 2021). Further research should investigate how sleep quality and duration are valued in Engineering culture and explore the role of gender.

As expected, Health students had the least dysfunctional attitudes toward sleep, aligning with Jin et al’s. (2018) speculation that medical education may help correct erroneous attitudes about sleep. Furthermore, their lower dysfunctional sleep attitudes were associated with better sleep quality and less insomnia severity (Yang and Chou, 2011; Calkins et al., 2012; Doos Ali Vand et al., 2014; Jin et al., 2018). Therefore, addressing dysfunctional attitudes about sleep may be a viable target for intervention. Indeed, cognitive behavioral therapy is a promising approach, as it has been shown to produce moderate to large reductions in dysfunctional sleep attitudes (Thakral et al., 2020), as well as improve sleep quality and reduce insomnia severity (van Straten et al., 2018).

In the present study, dysfunctional sleep attitudes were associated with longer sleep durations but worse sleep quality and insomnia severity. Theoretical frameworks suggest sleep may be impacted by an individual’s perceptions, behavioral intentions, and external factors (gender; Alamir et al., 2022). In the Alamir et al. (2022) study, perceptions of sleep were based on the Health Belief Model (HBM; Rosenstock, 1966), purporting that health behavior can be predicted by one’s perceptions of four variables: susceptibility, severity, benefits, and barriers. Within the HBM, perceived severity was positively associated with sleep duration while perceived barriers (e.g., feeling pressured for time, feeling behind on work) negatively predicted sleep duration in college students (Knowlden and Sharma, 2014). Prior research also suggests that self-efficacy plays a role, where students who feel in control of their time or sleep environment tend to have better sleep (Knowlden and Sharma, 2014; Knowlden and Naher, 2023). Considering our results in the context of these prior studies suggests that although knowing the negative consequences of poor sleep may help encourage good sleep behavior, other barriers including a lack of self-efficacy may reduce the impact that this knowledge has. Thus, having dysfunctional attitudes about sleep may increase a person’s motivation to improve their sleep, but those with time constraints, decreased self-efficacy, or extremely distressing/dysfunctional beliefs may still struggle to attain good quality sleep.

Education on sleep hygiene may be beneficial. Consistent with other studies, we found that poor sleep hygiene significantly predicted of sleep duration, sleep quality, and insomnia severity (Brown et al., 2002; Suen et al., 2008; Revathi et al., 2016; Ruggiero et al., 2019; Humphries et al., 2021; Shaheen and Alkaid, 2022; Ali et al., 2023). Sleep hygiene may act as a mediator between positive affect and sleep quality, suggesting that failing to engage in good sleep hygiene may exacerbate the bidirectional relationship between poor mental health and poor sleep (Li et al., 2016; Lukowski et al., 2019). Fortunately, sleep hygiene practices can be modified with education. For example, a program called “Sleep 101,” which consisted of two 90-min workshops covering general topics about sleep and sleep hygiene, alongside cognitive and behavioral strategies to improve sleep, significantly reduced sleep latency and dysfunctional sleep attitudes in college students (Kloss et al., 2016). Other education-based interventions for university students have been successful at improving sleep outcomes (Brown et al., 2006; Trockel et al., 2011; Hershner and O’Brien, 2018). These interventions demonstrate providing sleep programming may be an affordable and effective way for universities to support the well-being of their students and should be considered by policy makers and university offices.

There were several limitations to this study. First, there was unequal distribution of responses, as there was both over and under representation of students from different departments, and the sample was predominantly women/female, potentially biasing the results. A second limitation was that data collection occurred during the COVID-19 pandemic and the return to in-person learning which was shown to worsen student distress and mental health (Robillard et al., 2021; King et al., 2023). We tried to mitigate this by delaying the onset of the survey to several weeks after the return to in-person classes. Finally, the reliance on self-reported measures, especially regarding sleep, may impact the accuracy of the results. A strength of the study is the large sample size and diversity of students. The present sample consisted of a wide variety of academic disciplines, rather than homogenous (e.g., first year psychology students) or dichotomous (e.g., medical versus non-medical) groups. Additionally, 58% of participants identified as persons of color, demonstrating diversity across racial/ethnic groups. See Karsan et al. (2024) for racial/ethnic descriptions of this cohort.

## 5 Conclusion

To our knowledge, this is one of the few studies to examine sleep in university students across a wide array of distinct university disciplines. We found evidence that students from different academic disciplines have unique relationships with sleep quality and duration, but this may be impacted by differences in mental health, attitudes toward sleep, and sleep hygiene practices. Art students had significantly worse sleep quality, as well as mental health, highlighting that this student group may need additional supports for sleep and mental health. Identification of effective interventions to improve sleep and mental health is needed.

## Data availability statement

The raw data supporting the conclusions of this article will be made available by the authors, without undue reservation.

## Ethics statement

The studies involving humans were approved by University of Waterloo Office of Research Ethics #43903, McMaster University Research Ethics Board #5834. The studies were conducted in accordance with the local legislation and institutional requirements. The participants provided electronic informed consent to participate in this study.

## Author contributions

TK: Conceptualization, Data curation, Formal analysis, Investigation, Methodology, Project administration, Validation, Writing – original draft. SK: Writing – review & editing, Conceptualization, Data curation, Investigation, Methodology. JH: Conceptualization, Resources, Supervision, Writing – review & editing. LM: Conceptualization, Resources, Supervision, Writing – review & editing.
